# Beta-subunit-eliminated eHAP expression (BeHAPe) cells reveal subunit regulation of the cardiac voltage-gated sodium channel

**DOI:** 10.1016/j.jbc.2023.105132

**Published:** 2023-08-06

**Authors:** Annabel Y. Minard, Colin J. Clark, Christopher A. Ahern, Robert C. Piper

**Affiliations:** Department of Molecular Physiology and Biophysics, University of Iowa College of Medicine, Iowa City, Iowa, United States

**Keywords:** voltage-gated sodium channels, cardiac voltage-gated sodium channels, voltage-gated sodium channel β-subunits, eHAP cells, CRISPR/Cas9, electrophysiology, sodium channel, gene KO, gene mapping

## Abstract

Voltage-gated sodium (Na_V_) channels drive the upstroke of the action potential and are comprised of a pore-forming α-subunit and regulatory β-subunits. The β-subunits modulate the gating, trafficking, and pharmacology of the α-subunit. These functions are routinely assessed by ectopic expression in heterologous cells. However, currently available expression systems may not capture the full range of these effects since they contain endogenous β-subunits. To better reveal β-subunit functions, we engineered a human cell line devoid of endogenous Na_V_ β-subunits and their immediate phylogenetic relatives. This new cell line, β-subunit-eliminated eHAP expression (BeHAPe) cells, were derived from haploid eHAP cells by engineering inactivating mutations in the β-subunits SCN1B, SCN2B, SCN3B, and SCN4B, and other subfamily members MPZ (myelin protein zero(P0)), MPZL1, MPZL2, MPZL3, and JAML. In diploid BeHAPe cells, the cardiac Na_V_ α-subunit, Na_V_1.5, was highly sensitive to β-subunit modulation and revealed that each β-subunit and even MPZ imparted unique gating properties. Furthermore, combining β1 and β2 with Na_V_1.5 generated a sodium channel with hybrid properties, distinct from the effects of the individual subunits. Thus, this approach revealed an expanded ability of β-subunits to regulate Na_V_1.5 activity and can be used to improve the characterization of other α/β Na_V_ complexes.

Voltage-gated sodium (Na_V_) channels drive the upstroke of the action potential in excitable cells, thereby generating the electric signals that underlie behavior, sensation, muscle contraction, and mobility (1, 2, 3, 4). Na_V_ channels are composed of one pore-forming α-subunit and one or more regulatory β-subunits. The α-subunits are large (∼250 kDa) proteins with 24 transmembrane segments that are arranged in four domains (DI-DIV) to form a central sodium selective and conducting pore. The β-subunits are not required for conduction but instead function to regulate the α-subunit. Their functions include trafficking and retention of the α-subunit at the plasma membrane, altering the pharmacology of the α-subunit, and altering the voltage-dependent gating properties of the α-subunit (2, 5, 6). These functions are crucial for proper electrical conduction since even relatively small changes in the gating equilibria and rates between closed, open, and inactivated states can cause electrical dysfunction (7, 8). There are ten α-subunit isoforms and five Na_V_ β-subunit isoforms. The Na_V_ β-subunits, β1-4, are single-pass membrane proteins, with an extracellular immunoglobulin (lg)-like domain. These β-subunits are encoded by the genes SCN1B, SCN2B, SCN3B, and SCN4B. A fifth Na_V_ β-subunit, β1b, generated from a splice variant of SCN1B, is expressed as a secreted protein containing the Ig-like domain. The predominant Na_V_ channel in the heart is Na_V_1.5, which forms complexes with β1-β4 (9, 10, 11). Loss of Na_V_1.5 in mice is lethal *in utero* while loss of β1- β3 causes cardiac arrhythmia (12, 13, 14). Furthermore, clinical variants in Na_V_1.5 or β1-β4 are associated with cardiac arrhythmias and disorders such as Brugada Syndrome and Long QT syndromes (15, 16). Despite the importance of Na_V_1.5 and β-subunits, precisely and systematically defining the effect of β-subunits and their diseases variants on the electrophysiology of Na_V_1.5 has been challenging due in part to the limitations of currently available expression systems (17).

The electrophysiological properties of Na_V_ channels are routinely determined in heterologous systems, such as human embryonic kidney (HEK)-293T, CHO, and COS cells. These systems lack endogenous voltage-gated ion channels, allowing for the precise measurement of action-potentials generated by ectopically expressed α-subunits. However, they express endogenous β-subunits, which could alter α-subunit properties and interfere with the response to ectopically expressed β-subunits. To circumvent this issue *Xenopus laevis* oocytes are sometimes used since they have minimal endogenous β-subunit expression (18, 19). But because *X. laevis* oocytes require a low incubation temperature (18 °C), they are not ideal for the study of disease variants. In addition to endogenous β-subunits, the presence of phylogenetic relatives of β-subunits may also modify a channel of interest. The β-subunits belong to a subfamily of proteins (β/myelin protein zero [MPZ]) that includes MPZ, MPZL1, MPZL2, MPZL3, and JAML (20, 21). Although not implicated in directly regulating Na_V_ channels, these proteins may regulate the channel in cultured cells if they share biophysical properties with β-subunits. Indeed, the sequences of β1 and β3 are as similar to the MPZ subfamily as they are to β2 and β4 (20). Thus, an ideal expression system for studying Na_V_ β-subunit isoforms and their disease-associated alleles should lack an endogenous β/MPZ family.

In this study, we used CRISPR-Cas9 gene-editing to generate **β-**subunit-eliminated **eHAP** **e**xpression (BeHAPe) cells, a cell line devoid of the β/MPZ gene family. This cell line allows β-subunits to be expressed as the sole β-subunit family member without interference from endogenous β-subunits. We used these cells to assess the effects of β-subunits on the gating properties on the predominant cardiac α-subunit Na_V_1.5. We found that in BeHAPe cells Na_V_1.5 was highly responsive to β-subunit effects and that co-expression with β-subunits generated a repertoire of Na_V_1.5 channels with unique gating properties. These findings demonstrate that a β/MPZ null cell model improves the characterization of the electrophysiological properties of α/β Na_V_ complexes.

## Results

### Generation of BeHAPe cells

To better reveal how β-subunits modulate the gating properties of α-subunits, we generated a cell line devoid of the β-subunit family (SCN1B, SCN2B, SCN3B, and SCN4B) and their phylogenetic relatives (MPZ, MPZL1, MPZL2, MPZL3, and JAML). We deleted these genes from human haploid (eHAP) cells. eHAP cells are fibroblast like cells that do not produce endogenous voltage-gated sodium currents. These cells are also haploid, simplifying mutagenesis and genotyping. Previous RNAseq experiments (Accession: SRX655513, SRX65551) show that this cell line has low to moderate expression of β/MPZ family members (22). No expression of SCN2B or JAML was detected, whereas SCN1B, SCN3B, SCN4B, MPZ, MPZL1, MPZL2, and MPZL3 had Fragments Per Kilobase of transcript per Million mapped reads values of 0.70, 0.20, 0.016, 2.15, 3.40, 0.01, and 0.30, respectively, comparable to those in HeLa and HEK-293 cells (SRR1567907 and SRR5011299).

**Figure 1:**
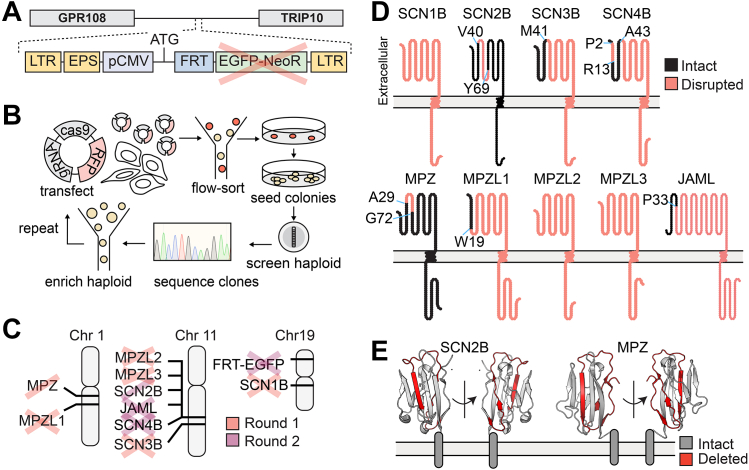
**Generation of BeHAPe cells by two rounds of CRISPR-Cas9 deletion of β-subunits and their phylogenetic relatives.***A*, eHAP-FRT cells have an FRT integration site between GPR108 and TRIP10. EGFP-NeoR was originally expressed from this locus but deleted in the second round of CRISPR-Cas9 deletions. *B*, eHAP-FRT cells were engineered into BeHAPe cells with two rounds of CRISPR-Cas9. Multiple gRNA, Cas9, and fluorescent marker encoding plasmids were transfected into the eHAP-FRT cell-line. Transfected cells were enriched for by flow-sorting for fluorescent cells and serially diluted onto 10 cm dishes. Clones were screened to verify haploidy and then genotyped. *C*, genes marked with a cross were disrupted in BeHAPe cells, while a single-line indicates the gene was edited but not with a frame-shifting mutation. Whether a gene was targeted in round one or round two are indicated. *D*, amino acid residues that are no-longer encoded for in the edited genes are shown. Protein sequence was visualized with Protter (62). *E*, residues deleted in SCN2B and MPZ genes encoded for a β-strand in the Ig domain (SCN2B PDB:5FDY and MPZ PDB: 30AI). BeHAPe, β-subunit-eliminated eHAP expression cells; CRISPR, clustered regularly interspaced short palindromic repeat; EGFP, enhanced green fluorescent protein; FRT, Flp recombination target site; gRNA, guide RNA; Ig, immunoglobulin.

We first integrated an Flp recombination target (FRT) site into eHAP cells to allow for site-directed recombination in future studies (Fig. 1*A*). The FRT site was encoded in the pQCXIP FRT EGFP-Neo vector, which was transduced into eHAP cells by retrovirus. We mapped the integration site to chromosome 19, in between GPR108 and TRIP10 genes. The introduced locus expressed enhanced green fluorescent protein (EGFP) fused to neomycin resistance gene (EGFP-Neo^R^) under a cytomegalovirus (CMV) promoter (pCMV). An FRT site was placed between the start codon and EGFP. Later, in the second round of CRISPR-Cas9 deletions, we deleted EGFP-Neo^R^, leaving the CMV promoter and FRT site available for optional use.

We next disrupted the β/MPZ family in eHAP-FRT cells in two rounds of CRISPR-Cas9 gene editing (Fig. 1*B*). For these experiments, Cas9 and guide RNA (gRNA) encoding plasmids, co-expressing fluorescent reporter proteins, were transiently transfected into cells. To edit nine genes in two rounds of CRISPR-Cas9, we introduced multiple gRNA per gene and targeted multiple genes in each round. To improve the efficiency of gene edits, we enriched haploid cells prior to transfecting gene editing reagents, which was necessary as eHAP cells diploidize. After transfection, we also enriched for fluorescent transfected cells by flow-actuated cell sorting (FACS) because transfection efficiency was roughly 30%. We identified KOs by PCR amplifying and Sanger sequencing the targeted genes. Because eHAP cells are haploid, they possess only one copy of the targeted genes. KOs were selected from cells with indel mutations that frame-shifted the protein coding sequence or removed the start codon. At some loci large insertions or repetitive sequences were introduced that could only be partially mapped by Sanger sequencing. To confirm these modifications, we also performed whole-genome sequencing using Oxford Nanopore MinION sequencing. This generated 1 to 3 reads across some of the modified loci that confirmed their genomic rearrangements but was not deep enough for precise base calling.

After the first round of mutagenesis, we generated a cell line (clone 25) that contained frame-shifting indel mutations in MPZL1, MPZL3, and SCN3B, deletion of the start codon of SCN1B, and an in-frame deletion of MPZ (Fig. 1*C*). Clone 25 was subject to a second round of mutagenesis, and this led to the generation of BeHAPe cells. BeHAPe cells contain additional frame-shifting indel mutations in SCN4B, JAML, and EGFP, an in-frame deletion of SCN2B and a larger in-frame deletion of MPZ.

The impact of the gene disruptions at the protein level are shown in Figure 1*D*. The gene-edits completely disrupted the protein coding sequences of SCN1B, MPZL2, and MPZL3, while the protein coding sequences of SCN3B, SCN4B, and MPZL1 encode only a truncated N terminus. While the deletion in MPZ left the codons for the signal sequence (M1-A29) intact, it removed I30-E71, which encodes for a central β-strand in the Ig domain (Fig. 1*E*). This deletion should render the Ig domain of MPZ misfolded and possibly subjected to ER-associated degradation, as is the fate of MPZ mutants associated with Charcot-Marie-Tooth disease (21). Similarly, SCN2B was deleted of V40-Y69, which encodes a central β-strand in the Ig domain and thus should also render the protein misfolded.

**Figure 2:**
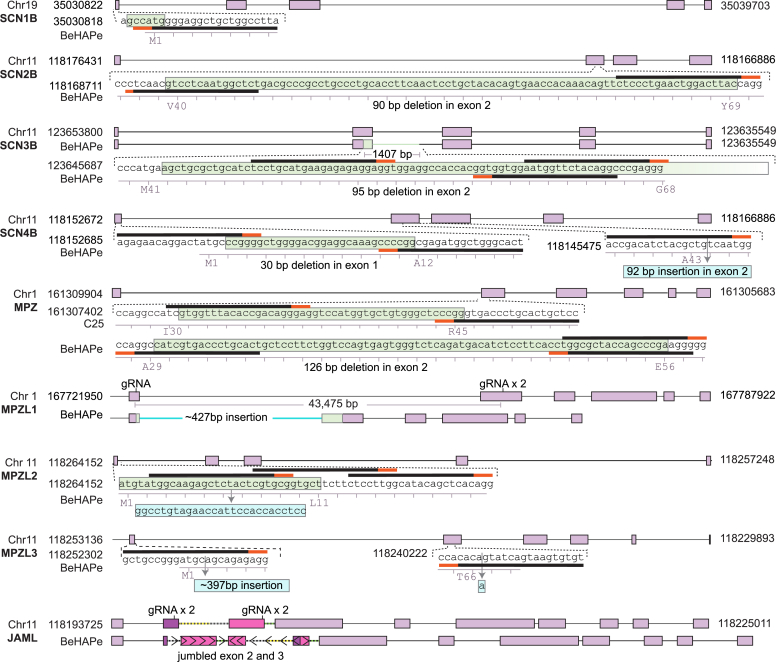
**Mutations in β-subunits and their phylogenetic relatives in BeHAPe cells.** Exons are drawn as *purple boxes*, deleted regions are colored *green*, and insertions are colored *cyan*. gRNA binding sites are indicated with a *black* and *red bar*, *red bar* indicates PAM sequence. The position of gene loci are derived from Genome Reference Consortium Human Build 38. BeHAPe, β-subunit-eliminated eHAP expression cells; gRNA, guide RNA; PAM, protospacer adjacent motif.

The edited genomic loci in BeHAPe cells are included in Supporting Information and are illustrated in Figure 2. In SCN1B, exon 1 had a 6 bp deletion that included the start codon. In SCN2B, exon 2 had an in-frame 90 bp deletion. In SCN3B, 1407 bp were deleted including a frame-shifting 95 bp from 3′ end of exon 2. In SCN4B, exon 1 had an in-frame 30 bp deletion and exon 2 had a frame-shifting 92 bp deletion. In, MPZ exon 2 had an in-frame 126 bp deletion. In MPZL1, a large 43,475 bp region that included portions of exon 1 and 2 was deleted and replaced with ∼427 bp. In MPZL2, a frame-shifting 32 bp from exon 1, which included the start codon, was replaced with 27 bp which kept the protein coding sequence out of frame. In MPZL3, ∼397 bp of repetitive sequences was inserted just after the encoded initiating Met. There was also a single base pair insertion in exon 2, causing a frameshift mutation. In JAML, exon 2 had a frame-shifting ∼2888 bp insertion. The JAML locus could not be genotyped with Sanger sequencing but was genotyped using long read whole-genome sequencing. This revealed that the inserted DNA was a partial duplication of intron 2 and exon 3 followed by a reversed exon 3, intron 2 and exon 2.

Surprisingly, we only recovered in-frame deletions of MPZ. In the first round of mutagenesis only a single clone (Clone 25) with an edited locus was recovered, and its locus had an in-frame deletion (Fig. 2). In the second round of mutagenesis several clones with further edited MPZ were recovered, yet all had in-frame edits. This included three clones with in-frame deletions; one clone with a frameshift deletion, which was corrected with a second frameshift deletion that restored the WT reading frame; and two clones that had replaced sequences with new sequences that preserved the reading frame. All these deletions left the signal sequence intact, and the largest span of deleted residues was between I30-E71. These data suggest that this protein may be essential in haploid eHAP cells. If so, the essential function it serves must not require the extracellular Ig domain since this domain was disrupted in BeHAPe cells. The major known function of MPZ is in the compaction of myelin, a function irrelevant to eHAP viability. In addition, MPZ KO mice are viable showing MPZ is not essential in multiple cell types (23).

### Patch-clamping BeHAPe cells

We next sought to examine the electrophysiological properties of the cardiac Na_V_ channel Na_V_1.5 in BeHAPe cells; however, whole cell patch-clamp experiments were initially challenging. BeHAPe cells were small, exhibited a low transfection efficiency, and a plasma membrane that was prone to blebbing, which made it difficult to obtain tight giga-Ohm seals. This plasma membrane morphology was not observed in eHAP cells, which were easier to patch-clamp. These problems were partially mitigated through the following optimization steps.

**Figure 3:**
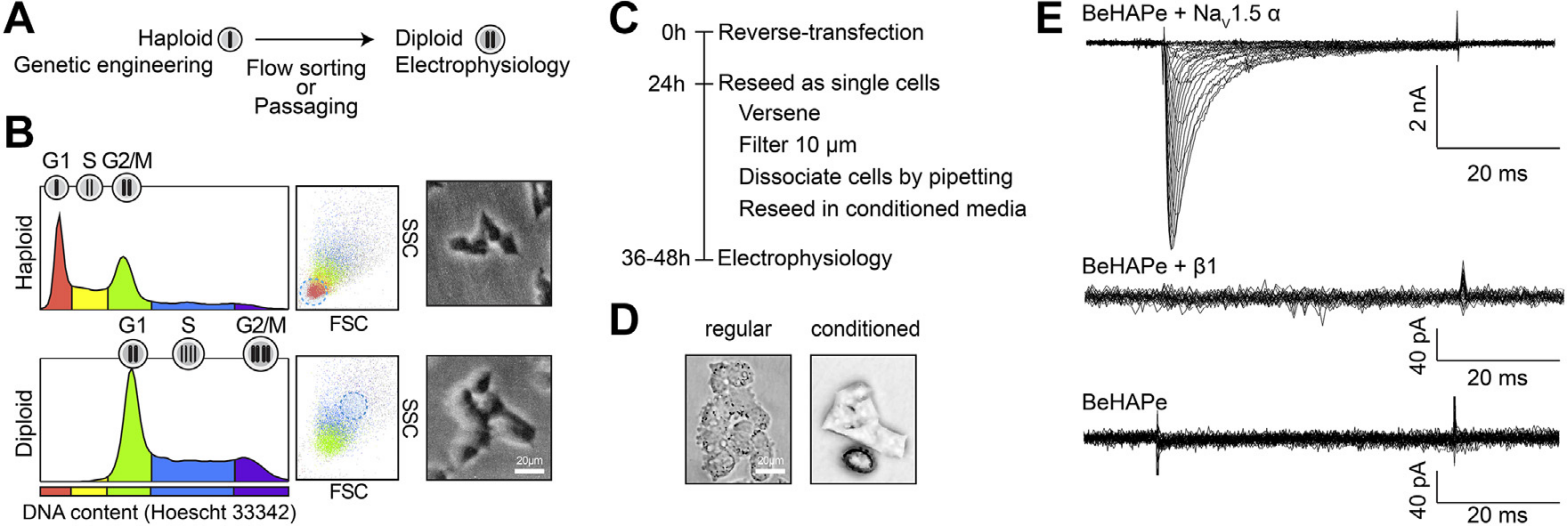
**Preparation of BeHAPe cells for electrophysiology.***A*, haploid cells were used for gene editing, while diploid cells were used for electrophysiology experiments. Haploid cells become spontaneously diploid after several passages. *B*, haploid or diploid BeHAPe cells can be enriched for by flow sorting based on cell size. BeHAPe cells were stained with Hoechst 33342 and DNA content was compared to forward (FSC) and side (SSC) scatter by flow cytometry. A micrograph of sorted haploid and diploid cells are shown. *C*, diploid BeHAPe cells were prepared for electrophysiology as follows. Cells were “reverse”-transfected and 24 h later reseeded as single cells. To obtain healthy single cells, cells were lifted with versene, resuspend in conditioned media, passed through a 10 μm mesh, and dissociated further with gentle pipetting. Thirty-six to forty-eight h after transfection cells were phenotyped with electrophysiology. *D*, BeHAPe cell membranes became blebby and incompatible with patch-clamping when resuspended in fresh media, however, remained smooth in conditioned media. *E*, sodium current recordings from BeHAPe cells untransfected or transfected with β1 or Na_V_1.5 cDNA, as indicated. BeHAPe, β-subunit-eliminated eHAP expression cells; cDNA, complementary DNA; Na_V_, voltage-gated sodium.

First, while haploid BeHAPe cells (and parental eHAP cells) are small, diploid BeHAPe cells are larger and easier to patch-clamp (Fig. 3). We obtained a diploid population by passaging haploid cells, which spontaneously diploidize, and sorting for diploid cells by FACS based on forward scatter (FSC) and side-scatter (SSC) profiles (24). We confirmed that the magnitude of FSC and SSC corresponded to haploid and diploid eHAP cells by staining DNA content in live cells using Hoescht 33342 (Fig. 3*B*). As Hoescht 33342 was toxic to cells, for routine sorting we set FSC and SSC gates using unstained reference populations.

Second, we achieved the highest expression of Na_V_1.5, with ∼30% transfection efficiency in BeHAPe cells when they were “reverse” transfected. Cells were reseeded as single cells 24 h after transfection and patch-clamped 12 to 16 h later, when there was peak expression of a tracer plasmid pmaxGFP (Fig. 3*C*). Since the expression of transgenes was variable, we analyzed only cells with similar levels of the GFP tracer and current density. This selection approach, however, precluded the use of peak current density as a proxy for trafficking of the α-subunit to the plasma membrane.

Finally, the plasma membrane of BeHAPe cells was prone to blebbing. The membrane blebbing was not suppressed by reexpression of β/MPZ subunits indicating a likely off-target effect. Plasma membrane blebbing could, however, be suppressed by growing BeHAPe cells in conditioned media and by avoiding sheer stress (Fig. 3*D*). To reseed cells as single cells prior to electrophysiology, we reduced the amount of pipetting, and thus shear stress, by passing cells through a 10 μm filter, which is approximately the diameter of a cell, and seeding dissociated cells in conditioned media. During patch-clamp experiments, the cells retained good morphology in external solution for 30 min.

These methods allowed whole cell patch-clamp experiments on BeHAPe cells. Like eHAP cells, BeHAPe cells show no voltage dependent currents when transfected with an empty plasmid vector or β-subunit (Fig. 3*E*). Robust currents were produced only after transfection with the α-subunit Na_V_1.5. Thus, this system can be used to reliably measure the electrophysiological properties of Na_V_ channels.

### Comparing electrophysiological effects of β1 on Na_V_1.5 in eHAP and BeHAPe cells

**Figure 4:**
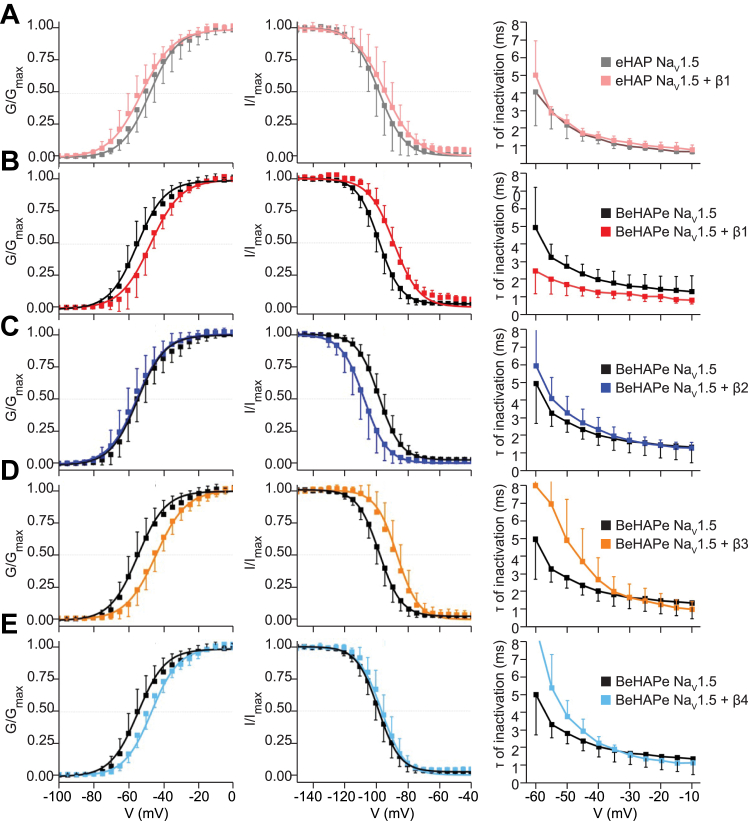
**Biophysical properties of Na**_**V**_**1.5 when co-expressed with β-subunits part 1.***A*, coexpression with β1 showed no effects on Na_V_1.5 biophysical properties in the parental eHAP cell line. Na_V_1.5 was expressed alone in eHAP cells and GV (n = 8), SSI (n = 6) and the rate of inactivation (n = 8) measured. When coexpressed with rat β1-V5-6xHIS, no significant change was found in GV V_1/2_ (n = 8, *p* = 0.77), SSI V_1/2_ (n = 6, *p* = 0.30), or the rate of inactivation at V_1/2_ of the GV (n = 8, *p* = 0.84). *B*–*E*, co-expression with β1-4 modulates Na_V_1.5 biophysical properties in the BeHAPe cells. Na_V_1.5 was expressed alone in BeHAPe cells and GV (n = 10), SSI (n = 9) and the rate of inactivation (n = 10) measured. Plots for Na_V_1.5 alone in BeHAPe are identical across graphs. *B*, rat β1-V5-6xHIS co-expression shifted Na_V_1.5 GV V_1/2_ (+6.7 ± 3.2 mV, n = 8, *p* = 0.02), SSI V_1/2_ (+8.8 ± 2.9 mV, n = 7, *p* = 0.0088), and rate of inactivation at V_1/2_ of the GV (−1.7 ± 1.0 ms, n = 8, *p* = 0.00025). *C*, human β2-HA co-expression shifted SSI V_1/2_ (−9.9 ± 3.1 mV, n = 6, *p* = 0.0019) but not GV V_1/2_ (n = 6, *p* = 0.33) or the rate of inactivation at V_1/2_ of the GV (n = 6, *p* = 0.30). *D*, human β3 co-expression shifted Na_V_1.5 GV V_1/2_ (+8.1 ± 3.4 mV, n = 7, *p* = 0.003) and SSI V_1/2_ (+9.1 ± 3.1 mV, n = 6, *p* = 0.0053), but not the rate of inactivation at V_1/2_ of the GV (n = 6, *p* = 0.34). *E*, human β4 co-expression shifted Na_V_1.5 GV V_1/2_ (+5.2 ± 3.0 mV, n = 6, *p* = 0.045) but not SSI V_1/2_ (n = 6, *p* = 0.46) or the rate of inactivation at V_1/2_ of the GV (n = 6, *p* = 0.30). *A*–*E*, data are plotted as the mean ± SD. For GV and SSI plots, mean values were fitted to Boltzmann functions. For rates of inactivation plots, rates were derived from a single exponential function manually fit to individual traces. NaV, voltage-gated sodium; SSC, side-scatter; SSI, steady-state inactivation.

To determine whether eliminating the endogenous β/MPZ genes rendered the BeHAPe cells more sensitive to β-subunit modulation of Na_V_ channels, we compared the electrophysiological properties of Na_V_1.5 with and without β1 in eHAP cells and BeHAPe cells (Fig. 4, *A* and *B*). The eHAP cells revealed no β1-subunit effects on conductance-voltage curves (GV), steady-state inactivation (SSI) curves or rates of inactivation of Na_V_1.5 (Fig. 4*A*). In BeHAPe cells, Na_V_1.5 without co-expressed β-subunits exhibited similar GV, SSI, and rate of inactivation at the V_1/2_ of the GV as in eHAP cells, but all were shifted upon β1 co-expression (Fig. 4*B*). Thus, these results demonstrate that eliminating the endogenous β/MPZ proteins unmasks the effects of exogenous β-subunits on Na_V_1.5.

### Electrophysiological effects of β/MPZ-proteins on Na_V_1.5 in BeHAPe cells

**Figure 5:**
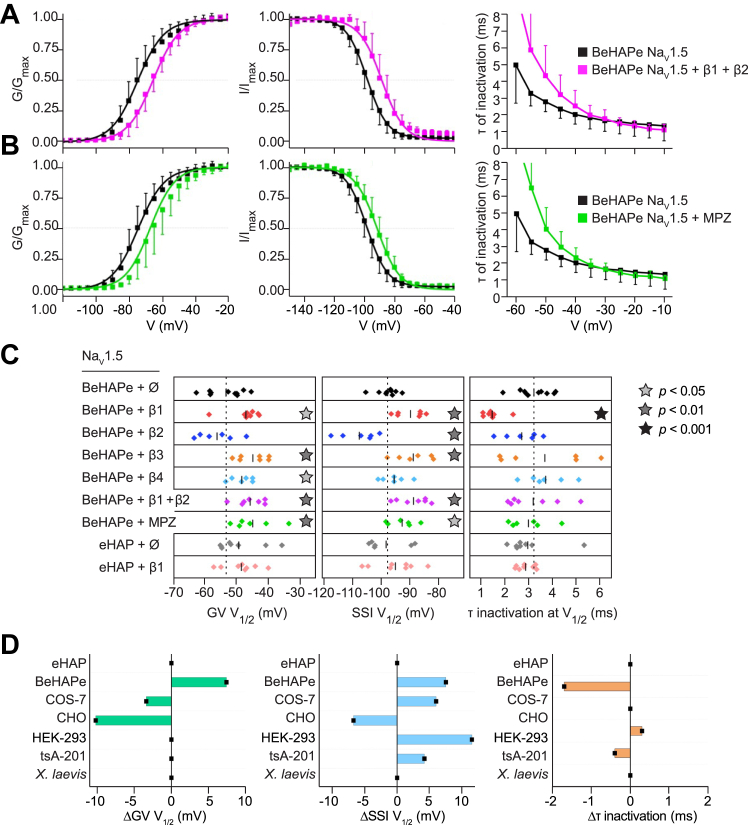
**Biophysical properties of Na**_**V**_**1.5 when co-expressed with β-subunits part 2.***A* and *B*, data for Na_V_1.5 alone in BeHAPe cells (n = 10 for GV, n = 9 for SSI, n = 8 for rate of inactivation) are identical to plots in Figure 4. *A*, simultaneous co-expression of both β1 and β2 shifted Na_V_1.5 GV V_1/2_ (+7.9 ± 3.1 mV, n = 7, *p* = 0.0073) and SSI V_1/2_ (+9.1 ± 3.0 mV, n = 7, *p* = 0.0031) but not the rate of inactivation at V_1/2_ of the GV (n = 7, *p* = 0.95). *B*, human MPZ co-expression shifted Na_V_1.5 GV V_1/2_ (+8.7 ± 3.3 mV, n = 6, *p* = 0.0048), SSI V_1/2_ (+6.3 ± 2.9 mV, n = 6, *p* = 0.035), but not the rate of inactivation at V_1/2_ of the GV (n = 6, *p* = 0.65). Data are plotted as the mean ± SD. For GV and SSI plots, mean values were fitted to Boltzmann functions. For rates of inactivation plots, rates were derived from a single exponential function manually fit to individual traces. *C*, summary of the gating properties of Na_V_1.5 alone or co-expressed with the indicated β/MPZ subunits. Shown are GV V_1/2_, SSI V_1/2_, τ of inactivation at V_1/2_ of the GV derived from the same experiments in Figure 4 and 5 *A* and *B*. Data points are individual replicates. *Black bars* represent mean values, which are reported in Table 1. *Star* indicates values were significantly different compared to Na_V_1.5 alone in BeHAPe cells as determined by a one-way ANOVA followed by Fisher’s LSD. *D*, comparison of the magnitude of β1 modulation of Na_V_1.5 recorded in eHAP and BeHAPe cells to previously reported values in other heterologous systems. Electrophysiological properties are plotted as the difference between Na_V_1.5 alone and Na_V_1.5 co-expressed with β1. Values are reported in Tables 1 and 2. BeHAPe, β-subunit-eliminated eHAP expression cells; LSD, least significance difference; NaV, voltage-gated sodium; SSI, steady-state inactivation.

We next used BeHAPe cells to compare the individual effects of all β-subunits (β1-4) on the gating properties of the major cardiac α-subunit Na_V_1.5 (Fig. 4 and Table 1). We also included MPZ in our analysis to gauge the capacity of β-subunit phylogenetic relatives to affect Na_V_1.5 gating (Fig. 5 and Table 1). The voltage dependence of activation (GV), SSI, and rate of inactivation curves are plotted in Figures 4 and 5. A summary of the gating properties with individual replicates are displayed in Figure 5*C* and the values obtained are recorded in Table 1. Remarkably, the β-subunits and MPZ all modulated at least one electrophysiological property of Na_V_1.5.

**Table 1:** Gating properties of Na_V_1.5 in BeHAPe cells

Channel	GV				SSI				τ inactivation (at V_1/2_)		
	V_1/2_ (mV)	ΔV_1/2_ (mV)	k (Slope)	N	V_1/2_ (mV)	ΔV_1/2_ (mV)	k (Slope)	N	τ (ms)	Δτ (ms)	N
eHAP Na_V_1.5	−49.8 ± 7.1	--	8.2 ± 0.4	8	−98.4 ± 7.3	--	7.0 ± 0.4	6	3.0 ± 0.9	--	8
eHAP Na_V_1.5 + β1	−53.4 ± 5.5	0.2 ± 3.3 (*p* = 0.77)	8.0 ± 0.3	8	−94.6 ± 7.7	3.2 ± 3.4 (*p* = 0.30)	7.4 ± 0.3	8	2.9 ± 0.4	−0.3 ± 1.0 (*p* = 0.839)	8
BeHAPe Na_V_1.5	−53.6 ± 5.6	--	7.9 ± 0.4	10	−97.8 ± 3.6	--	6.8 ± 0.2	9	3.2 ± 0.6	--	10
BeHAPe Na_V_1.5 + β1	−46.9 ± 4.9	6.7 ± 3.2∗ (*p* = 0.020)	8.0 ± 0.4	8	−89.0 ± 4.9	8.8 ± 2.9∗∗ (*p* = 0.0088)	7.1 ± 0.3	7	1.5 ± 0.4	−1.7 ± 1.0∗∗ (*p* = 0.00025)	8
BeHAPe Na_V_1.5 + β2	−56.1 ± 6.1	−2.5 ± 3.4 (*p* = 0.33)	8.2 ± 0.5	6	−107.7 ± 6.5	−9.9 ± 3.1∗∗ (*p* = 0.0019)	6.4 ± 0.3	6	2.7 ± 0.7	−0.5 ± 1.1 (*p* = 0.30)	6
BeHAPe Na_V_1.5 + β3	−44.8 ± 4.6	8.8 ± 3.1∗∗ (*p* = 0.0030)	7.4 ± 0.9	7	−88.7 ± 6.3	9.1 ± 3.1∗∗ (*p* = 0.0053)	7.2 ± 0.5	6	3.7 ± 1.8	0.4 ± 1.5 (*p* = 0.34)	6
BeHAPe Na_V_1.5 + β4	−48.4 ± 3.4	5.2 ± 3.0∗ (*p* = 0.045)	8.0 ± 0.3	6	−95.4 ± 4.4	2.4 ± 2.8 (*p* = 0.46)	7.0 ± 0.4	6	3.7 ± 0.9	0.5 ± 1.2 (*p* = 0.30)	6
BeHAPe Na_V_1.5 + β1 & β2	−45.7 ± 4.3	7.9 ± 3.1∗∗ (*p* = 0.0073)	8.1 ± 0.4	7	−88.5 ± 5.4	9.1 ± 3.0∗∗ (*p* = 0.0031)	7.2 ± 0.4	7	3.2 ± 1.1	0.0 ± 1.3 (*p* = 0.95)	7
BeHAPe Na_V_1.5 + MPZ	−44.9 ± 6.5	8.7 ± 3.3∗∗ (*p* = 0.0048)	7.4 ± 0.3	7	−91.5 ± 4.7	6.3 ± 2.9∗ (*p* = 0.035)	7.4 ± 0.3	6	3.0 ± 0.8	0.2 ± 1.2 (*p* = 0.65)	6
eHAP Na_V_1.5	−49.8 ± 7.1	3.8 ± 3.5 (*p* = 0.13)	8.2 ± 0.4	8	−98.4 ± 7.3	−0.6 ± 3.3 (*p* = 0.85)	7.0 ± 0.4	6	3.0 ± 0.9	−0.2 ± 1.2 (*p* = 0.57)	8
eHAP Na_V_1.5 + β1	−53.4 ± 5.5	0.2 ± 3.3 (*p* = 0.80)	8.0 ± 0.3	8	−94.6 ± 7.7	3.2 ± 3.4 (*p* = 0.44)	7.4 ± 0.3	8	2.9 ± 0.4	−0.3 ± 1.0 (*p* = 0.21)	8

Data are reported as mean ± SD. One-way ANOVA followed by Fisher’s least significance difference was used to compare data to Na_V_1.5 alone in either eHAP (top) or BeHAPe (bottom); ∗*p* < 0.05, ∗∗*p* < 0.01. Data in this table were used to generate Figures 4 and 5. Values of V_1/2_ for GV and SSI as well as slopes were extracted from Boltzmann fits to mean values. Rates of inactivation were derived from a single exponential function manually fit to individual traces.

Co-expression with β1 in BeHAPe cells shifted the midpoint of the GV and SSI curves of Na_V_1.5 to more positive potentials (+6.7 ± 3.2 mV for GV and +8.8 ± 2.9 mV for SSI) without altering the slope of these curves (Fig. 4*B*). β1 also increased the exponential rate of fast inactivation of Na_V_1.5 and was the only β-subunit to affect this property (−1.7 ± 1.0 ms) (Figs. 4*B* and 5*D*).

β2 was the only β-subunit to shift the midpoint of the SSI curve to more negative potentials (−9.9 ± 3.1 mV), and it did not shift the GV curve or the slope of either curve (Fig. 4*C* and Table 1). The effect of β3 on GV and SSI was almost identical to β1, with β3 shifting the midpoint of the GV and SSI curves to more positive potentials (+8.1 ± 3.4 mV for GV and +9.1 ± 3.1 mV for SSI) without altering the slope or the rate of fast inactivation (Fig. 4*D* and Table 1). Unlike the other β-subunits, β4 had no effect on the SSI curve but did shift the midpoint of the GV curve to more positive potentials (+5.2 ± 3.0 mV shift in GV) (Fig. 4*E*).

Since β1 and β2, when individually expressed, had opposing effects on Na_V_1.5 gating, we examined the effect on Na_V_1.5 gating properties when both were expressed simultaneously (Fig. 5*A*). In contrast to the effects of β1, expressing both β1 and β2 did not significantly change inactivation rates, but did shift the midpoint of the GV and SSI curves to more positive potentials (+7.9 ± 3.1 mV shift in GV, +9.1 ± 3.0 mV shift in SSI) (Fig. 5*A* and Table 1). These data indicated the effects of β1 override those of β2 on equilibrium gating, but the presence of β2 negated the effects of β1 on inactivation kinetics.

We were surprised to find substantial effects on Na_V_1.5 gating by MPZ, which, like β1 and β3, shifted the midpoint of the GV and the SSI curves to more positive potentials (+8.7 ± 3.3 mV for GV and +6.3 ± 2.9 mV for SSI) but, as with β2-4, did not affect rates of fast inactivation (Fig. 5*B*). Overall, these data demonstrate that each of the β-subunits and their phylogenetic relative MPZ have unique effects on Na_V_1.5 gating parameters, and that BeHAPe cells can be used as a platform for revealing these effects.

## Discussion

Na_V_s produce electrical signals in excitable cells, and dysfunction in the channel’s pore-forming α-subunit or auxiliary β-subunits are associated with a range of conduction diseases (7, 25, 26, 27, 28). β-subunits modulate the gating properties of α-subunits (2). Despite its importance, this function is difficult to study with currently available expression systems. Notably, the effects of β-subunits vary across expression systems as exemplified in Table 2 and Figure 5*D* (8, 9, 11, 29, 30, 31, 32, 33, 34, 35, 36, 37, 38, 39, 40). We hypothesized that the effects of ectopically expressed β-subunits could be better resolved in an expression system without endogenous β-subunits and their phylogenetic relatives, the MPZ-related proteins. In support of this hypothesis, we found that eliminating β/MPZ genes from eHAP cells sensitized Na_V_1.5 to modulation by ectopically-expressed β1. We used the newly generated BeHAPe cells to evaluate the effects of β1-4 on the electrophysiological properties of the main cardiac voltage-gated sodium channel, Na_V_1.5. We observed that each of the β-subunits uniquely modulated the Na_V_1.5 channel and the effects we obtained were generally different than observed in previous studies. These findings demonstrate the benefit of using expression systems devoid of endogenous β-subunits in basic studies on α/β interactions and may also be beneficial in studies on Na_V_ pharmacology and disease-causing mutations.

**Table 2:** Gating properties of Na_V_1.5 in previous studies

Expression system	Channel	GV		SSI		τ inactivation (ms at −40 mV)		Publication
		V_1/2_ (mV)	ΔV_1/2_ (mV)	V_1/2_ (mV)	ΔV_1/2_ (mV)	τ (ms)	Δτ (ms)	
*X. laevis*	Na_V_1.5	−40.6 ± 1.2		−69.2 ± 0.8		0.7 ms		(44)
	Na_V_1.5 + β1	−40.6 ± 1.9	NS	−70.5 ± 0.8	NS	0.7 ms	NS	
*X. laevis*	Na_V_1.5	−18.6 ± 4.2		−65.3 ± 0.9		NR		(54)
	Na_V_1.5 + β1	−23.3 ± 2.0	NS	−65.9 ± 0.7	NS	NR		
	Na_V_1.5 + β3	−21.3 ± 2.4	NS	−60.7 ± 1.3	5.2	NR		
tsA-201	Na_V_1.5	−25.6 ± 1		−77.1 ± 0.5		NR		(9)
	Na_V_1.5 + β1	−24.9 ± 1	NS	−72.9 ± 1.0	4.2	NR		
	Na_V_1.5 + β2	−23.2±1	2.4	−77.1±1.1	NS	NR		
	Na_V_1.5 + β1 + β2	−24.3±2	NS	−74.0 ± 1.0	3.1	NR		
tsA-201	Na_V_1.5	−35.4 ± 1.2		−88.2 ± 0.9		NR		(45)
	Na_V_1.5 + β1	−34.7 ± 1.2	NS	−81.8 ± 0.9	NS	NR		
HEK-293T	Na_V_1.5	−37.8 ± 1.6		−95.2 ± 2.9		2.3		(46)
	Na_V_1.5 + β1	−36.7 ± 0.6	NS	−90.9 ± 2.1	4.3	1.9	−0.4	
HEK-293T	Na_V_1.5	−43.78 ± 1.28		−78.77 ± 1.11		0.68 (−20 mV)		(11)
	Na_V_1.5 + β4	−43.69 ± 1.61	NS	−82.28 ± 0.74	−3.5	0.77 (−20 mV)	NS	
HEK-293	Na_V_1.5	NR		−70.2 ± 1.3		NR		(8)
	Na_V_1.5 + β1	−22		−58.7 ± 1.2	11.5	1.1 ms		
CHO	Na_V_1.5	−38.7 ± 0.5		−86.9 ± 1.5		NR		(43)
	Na_V_1.5 + β1	−48.9 ± 0.8	−10.2	−93.6 ± 0.8	−6.7	NR		
	Na_V_1.5 + β2	−38.0 ± 0.6	NS	−80.2 ± 0.6	6.7	NR		
CHO-K1	Na_V_1.5	−16.2 ± 1.0		−61.7 ± 1.9		2.0 ms		(47)
	Na_V_1.5 + β1	−22.2 ± 1.8	−6	−68.7 ± 2.5	−6.7	2.3 ms	0.3	
COS-7	Na_V_1.5	−33.5 ± 0.3		−95.8 ± 0.3		1.2 ms		(41)
	Na_V_1.5 + β1	−36.1 ± 0.6	−3.4	−89.8 ± 0.2	6	1.1 ms	NS	

Values are presented as mean ± SEM. Data in this table were used to generate Figure 5*D*.

The study of Nav channels in heterologous systems remains a valuable approach for the precise measurement of channel electrophysiological properties. By removing the channel from its native context, currents generated only by a channel of interest can be studied. However, the ability to study β-subunit effects on Na_V_ channels may be limited in currently available systems since they express endogenous β-subunits and their phylogenetic relatives. The effects of β1 we observed in BeHAPe cells were different from those in similarly conducted studies in human embryonic kidney 293 HEK-293, COS-7, and CHO cells, and X. laevis oocytes (Fig. 5*D* and Table 2) (8, 9, 11, 29, 30, 31, 32, 33, 34, 35, 36, 37, 38, 39, 40). Notably the effects of β1 on the voltage-dependence of activation observed in BeHAPe cells have not been observed in *X. laevis* oocytes or HEK-293 cells, and the effects on the voltage-dependence of inactivation have not been observed in *X. laevis* oocytes (29, 31, 32, 41). Furthermore, the effect of β1 on the rate of Na_V_1.5 inactivation was large compared to other systems. Na_V_1.5 produced a voltage-gated sodium current with a rate of fast inactivation that was relatively slow compared to previously published values in other expression systems (2.0 ms at −40 mV). However, when co-expressed with β1, the inactivation rate increased to values similar to those seen in other systems (1.3 ms at −40 mV). These results suggest that the BeHAPe cells provide a sensitive platform for revealing β-subunit effects on electrophysiological properties of Na_V_ channels. Our findings also show the complications that endogenous expression of β-subunits can pose. Notably all the β-subunits regulated the Na_V_1.5 channel, as did the structurally related protein MPZ. Thus, to avoid interference from endogenous β-subunits, the entire family should be removed.

There are many Na_V_ channels for which the precise electrophysiological properties are sought after, and thus would be best resolved in a clean β/MPZ null background. These include the nine other α-subunits that may have different responses to the β-subunits, and clinical variants of α and β-subunits. However, phenotyping numerous α/β channels in BeHAPe cells is difficult because the cells are not compatible with high-throughput screening. BeHAPe cells are not as robust as standard cell lines and require careful preparation for transfection and to avoid membrane blebbing. For these studies it would be beneficial to engineer a more robust cell line, which might be achieved by deleting β/MPZ proteins from, for example, HEK-293 cells. This heterologous expression system could help ascertain the electrophysiological properties of Na_V_ channels and thereby complement studies in differentiated pluripotent stem cells or primary cells that examine Na_V_ channels in a more complex native environment.

Despite the clear association of Na_V_1.5 with β-subunits, the structural basis for association and modulation of the Na_V_1.5 α-subunit remains unknown (42, 43). We found that the β-subunits each elicited a unique combination of effects on the GV, SSI, and kinetics of inactivation on Na_V_1.5. Interestingly the β-subunits most similar in sequence exerted similar effects. β1 and β3 both shifted the GV and SSI to more positive voltages, whereas β2 and β4 did not. MPZ, which is similar in sequence to β1 and β3, also shifted the GV and SSI to more positive potentials. Although not a physiological interactor of Na_V_1.5, MPZ can be used as a structural tool for gaining insight into the molecular basis of the differential effects of the β-subunits. We also found that the combination of β1 and β2 modulated Na_V_1.5 in ways that were distinct from the effects of individual subunits (Table 1). Some Na_V_ α-subunit isoforms are known to be capable of binding multiple β-subunits simultaneously (44, 45). Na_V_1.5 may have this property as well, allowing it to form a hybrid complex with different properties. Further experiments in a cell background such as the BeHAPe cells now enable the nuanced regulatory effects of β-subunits to be dissected.

The unique effects of β1-β4 on Na_V_1.5 gating properties could play a major role in cardiac rhythm. Although the specific function of β-subunits in heart is not defined, they are physiologically important since mutations in the subunits are associated with arrhythmias and KO of β-subunits (β1- β3) in mice cause cardiac arrhythmia (12, 13, 14). Intriguingly, the β-subunits are reported to occupy different subcellular regions in cardiac myocytes and concentrate in different regions of the heart (9, 46, 47, 48, 49). It seems likely that β-subunits operate *in vivo* to fine-tune heart physiology by modulating Na_V_1.5 gating at different locales. Another intriguing property of Na_V_1.5 revealed here is that the inactivation kinetics of the α-subunit are accelerated by only β1. This property may be linked to forms of cardiac dysfunction that are accompanied by a persistent sodium current caused by altered inactivation kinetics (50, 51, 52). If so, altered association with β-subunits within a subpopulation of Na_V_1.5 complexes might contribute to such a late current. We also observed that MPZ regulated Na_V_1.5, and although this is unlikely to be a physiological interaction given that MPZ is not expressed in the heart (53), there is a possibility that MPZ family proteins do regulate Na_V_ channels physiologically. For example, MPZL2 and MPZL3 are expressed in cardiac myocytes, and SCN5A is expressed in other cells besides cardiac myocytes (proteinatlas.org) (54). The broader expression pattern of the β/MPZ-subunit family, and thus its potential for altering the behavior of other Na_V_ isoforms, remains to be explored.

Our strategy for studying the β-subunit family can also be employed for characterizations of other gene families. Genes within a family, such as Na_V_ β/MPZ-subunit family, can often compensate for each other due to their structural similarity. In these circumstances, a gene-of-interest might only reveal its full range of activity in an expression system devoid of all compensating isoforms. While deletion of multiple genes from an expression system was previously time consuming, we show that it can be done relatively efficiently in haploid eHAP cells. The disadvantage of transfecting multiple gRNAs simultaneously is that recovered clones may have off-target mutations such that BeHAPe cells may not be precisely congenic with its parent. For this reason, a gene of interest should always be characterized by reintroducing it into the new cell line, rather than by comparing the mutant cell line to the WT parental cells.

Besides Na_V_1.5, there are nine other α-subunits and each may be modulated differently by the β-subunits. There are also disease-causing mutations in α and β-subunits that may affect channel properties. Our studies in BeHAPe cells demonstrate that the consequences of these interactions are best defined in a clean β/MPZ null background. However, as the BeHAPe cells are only suited to low throughput analysis, a systematic high-throughput electrophysiology analysis would greatly benefit from the engineering of a robust β/MPZ null cell line.

## Experimental procedures

### Molecular biology reagents

Plasmids and oligonucleotides used in this study are described in Supporting Information (Table S1). All enzymes targeting DNA and RNA were obtained from New England Biolabs (Ipswitch).

### Cell culture

Human haploid eHAP cells (Cat #C669, Horizon Discovery) were cultured in Iscove's modified Dulbecco's medium (Gibco) supplemented with 10% fetal calf serum. Cells were passaged every 48 to 72 h. As noted previously, eHAP cells can diploidize and make aneuploid populations (24). Thus, eHAP cells and their derivates were enriched for haploid cells using FACS using size (FSC and SSC) as the sorting parameter. Haploid-enriched populations of eHAP cells were used for gene engineering, whereas diploid cells were used for electrophysiology experiments. For transfection, Lipofectamine 3000 (Invitrogen/Thermo Fisher Scientific) was used in a reverse-transfection scheme as described (55) using manufacturer’s instructions for making DNA-Lipofectamine particles. Some culture conditions required conditioned media which was made as follows: BeHAPe cells were seeded onto a 100 mm dish, allowed to become confluent, after which they were grown for an additional week. The media were then harvested and passed through a 0.22 μm filter prior to storage at 4 °C until used.

### Generation of BeHAPe cells

An FRT site was first integrated into eHAP cells. eHAP cells were transduced with lentivirus carrying the pQCXIP FRT EGFP-Neo^R^ (pPL6490) vector. pPL6490 encodes an FRT site, GFP, and neomycin-resistance. pPL6490 was derived from pQCXIP (56), a bicistronic retroviral expression vector that originally conferred puromycin-resistance. pPL6490 was packaged into lentivirus in HEK-293 cells using the previously described lentivirus packaging system (57). Transduced eHAP cells were selected for by isolating neomycin-resistant colonies that express EGFP. From these clones we identified eHAP-FRT. The FRT integration site in eHAP-FRT was defined by isolating the integrated plasmid DNA and the genomic DNA flanking the integration site. This was achieved by first digesting genomic DNA with the restriction enzymes HindIII-HF, EcoRV-HF, HpaI, ApaI, BamHI-HF, XhoI, StuI, as well as RNAaseA and Klenow fragment in the presence of 1 mM dNTPs. Digested genomic DNA was ligated with T4 DNA ligase and electroporated into SURE bacterial cells (Agilent). Plasmids from ampicillin-resistant colonies were Sanger sequenced to define the FRT insertion described.

To disrupt genes encoding the β/MPZ family, two rounds of CRISPR-Cas9 gene-editing were performed on eHAP-FRT cells. Multiple gRNA sequences that targeted the N-terminal signal sequences or the exofacial Ig-like domains were used per gene. In round 1, eHAP-FRT cells were transiently transfected with pU6-(BbsI)_CBh-Cas9-T2A-mCherry (58) encoding *Streptococcus pyogenes* Cas9 and mCherry, and nine plasmids derived from pCLIP dual SFFV ZsGreen (Transomics) that encoded gRNAs against MPZ, MPZL1, MPZL3, SCN1B, and SCN3B (Table S1). After 48 h, ZsGreen and mCherry double positive cells were isolated by FACS as described below, and serially diluted into 100 mm plates and allowed to form colonies. Colonies were isolated using trypsin-soaked discs as previously described (30) and assessed for ploidy using propidium iodide staining. Haploid populations were genotyped as described below. One clone (clone 25) had frame-shifting indel mutations in all targeted genes, although MPZ had an in-frame deletion. Clone 25 cells were FACS-sorted for haploid cells and then subjected to another round of CRISPR/Cas9 mediated mutagenesis targeting SCN2B, SCN4B, JAML, MPZ, and EGFP at the FRT locus. Here, gRNA encoding sequences were cloned into the BbsI site downstream of the U6 promoter of pU6-(BbsI)_CBh-Cas9-T2A-mCherry to generate 16 different plasmids. Clone 25 cells were transiently transfected with these 16 plasmids, and 48 h after transfection, cells positive for mCherry were isolated by FACS, serially diluted, and allowed to form colonies after plating. Clones were isolated on cloning discs, and after expansion were assessed for ploidy using propidium iodide staining and genotyped. One clone, BeHAPe cells, had frame-shifting indel mutations in all targeted genes, except for MPZ and SCN2B, which acquired a large in-frame deletion. BeHAPe cells were FACS-sorted for haploid cells before storage.

### Genotyping of eHAP-derived cells

Genomic DNA was harvested using proteinase K and phenol–chloroform extraction (59). The CRISPR-targeted genetic loci were PCR amplified from genomic DNA using NEBNext High-Fidelity 2X PCR Master Mix and the primer pairs in Table S1. PCR products were purified, and Sanger sequenced using the primers also listed in Table S1. We also performed whole genome sequencing using an Oxford Nanopore MinION equipped with a version 10.41 flow cell. Reads were mapped using miniMAP2 using the default parameter for Oxford Nanopore data (60). Sanger sequences of PCR amplicons and Oxford Nanopore DNA sequences of edited loci are included in Supporting Information as are the annotated sequences of the edited loci.

### Ploidy analysis and FACS

The ploidy of eHAP cells was determined by propidium iodide staining and analyzed on a Becton Dickinson LSR II flow cytometer. Cells were trypsinized, washed twice with PBS, lysed, and stained using Nicoletti buffer (0.1% sodium citrate, 0.1% Triton X-100, 0.5 unit/ml RNase A, 20 units/ml RNase T1, and 50 μg/ml propidium iodide). Haploid eHAP cells were used for reference. Ploidy was also assessed by Hoescht 33342 staining. Hoescht 33342 (5 μg/ml) was applied to live eHAP cells for 10 min, cells were then trypsinized, and fluorescence intensity measured on a Becton Dickinson Aria II. Cells showed strong correlation between cell size and Hoescht intensity allowing the former parameter to be used to enrich haploid cells *via* cell sorting as described (24).

For cell sorting experiments, a Becton Dickinson Aria II equipped with a 130 μM diameter nozzle was used. Haploid or diploid cells were enriched by gating based on FSC and SSC. Reference populations were used to define the gates. Cells transiently expressing mCherry and/or ZsGreen were sorted for by using gates that captured the brightest 0.3% of cells.

### Preparation of cells for electrophysiology experiments

Electrophysiology experiments were performed on diploid BeHAPe cells using “reverse” transient transfection of the plasmids listed in Table S1. All constructs were grown in DH5α cells (NEB #2987H), prepared using PureLink HiPrep plasmid preparation kits (Invitrogen/Thermo Fisher Scientific) followed by sequencing of the reading frame and promoter. All constructs expressed their respective ORF *via* the CMV promoter. Cells were transfected with plasmids expressing Na_V_1.5-V5, pmaxGFP expressing *Pontellina plumata* GFP (Amaxa Biosciences), and either empty vector (pcDNA3.1) or plasmid expressing a β/MPZ family member using a ratio of 2:1:2, respectively. Twenty-four hours after transfection, cells were dissociated from tissue culture dish using Versene (Gibco), washed and resuspended in conditioned Iscove's modified Dulbecco's medium with 10% fetal calf serum. Cells were then immediately passed through a 10 μM nylon net filter (Millipore) and dispersed further by gentle pipetting before plating in 35 mm corning cell culture dishes (SKU: CLS430165, Corning). Twelve to twenty-fourhours after reseeding cells, electrophysiology experiments were performed.

### Whole-cell voltage patch clamp

Ionic currents through Na_V_1.5 (SCN5A) channels expressed in BeHAPe cells were recorded using whole-cell patch on Axon Axopatch 200B amplifiers (Molecular Devices). Data were collected and analyzed with pClamp11/Clampfit11 (Molecular Devices) and Origin software (OriginLab; https://www.originlab.com/index.aspx?go=PRODUCTS/Origin). Glass microelectrodes had resistances of 1.5 to 2 M Ω. Internal solution consisted of 105 mM CsF, 33 mM NaCl, 10 mM Hepes, and 10 mM EDTA, pH-adjusted to 7.3 with CsOH. External solution contained 150 mM NaCl, 2 mM KCl, 1.5 mM CaCl_2_, 1 mM MgCl_2_, 10 mM Hepes, pH-adjusted to 7.4 with NaOH. Included recordings had currents between 1 nA and 12 nA, access resistance of <6 MΩ, and compensated series resistance of >90%. All cells were recorded 36 to 48 h posttransfection. Data from at least three separate transfections constituted each dataset. Cells of similar size were analyzed by recording only from cells with whole-cell capacitance between 5 to 10 pF. Data were sampled at 20 kHz and filtered at 5 kHz. Leak currents were subtracted with the P/8 protocol (61), except in the case of the β-subunit only control conditions wherein the minute leak current was not subtracted to ensure integrity of control measurements. The conductance-voltage relationship (GV) curves were determined by dividing the measured current amplitude at a given test voltage by the driving force. GV and SSI measurements were averaged, and the means fit to a Boltzmann function confined between 0 and 1 to produce experimental values. For rates of inactivation, all data were P/8-subtracted and currents were fit to a single exponential with the function y = A + A_0_exp^−t/t^, with the bounds of the fit currents set manually to include the fast inactivating portion of the trace. Comparison of these rates was performed at the V_1/2_ of the GV, respective to experimental condition, to ensure equivalence of analysis regardless of shifts in GV relationships. Multiple authors conducted blinded exponential fits ensure fit integrity. Example trace data for different conditions are provided in Supporting Information (Fig. S1).

### Statistical analysis

Statistical analyses were carried out using Origin Lab. Statistical significance was assessed with a one-way ANOVA with Fisher’s least significance difference *post hoc* test without correction for multiple comparisons. Data are presented as mean ± SD. Individual values are reported in Table S2 in Supporting Information.

## Data availability

BeHAPe cells are available upon request from Robert Piper or Chris Ahern, University of Iowa. Annotated sequence maps of BeHAPe genes that were mutated and the key sequence data used to determine the BeHAPe genotype are included in Supporting Information. All data used to generate the electrophysiology graphs and tables are also provided in Supporting Information.

## Supporting information

This article contains supporting information.

## Conflict of interest

The authors declare that they have no conflicts of interest with the contents of this article.

## Supporting information

Supporting Figure S1

beHAPe_MPZ.ab1

beHAPe_SCN2B.ab1

beHAPe_SCN4B - 1.ab1

beHAPe_SCN4B - 2.ab1

C25_MPZ.ab1

C25_MPZL2.ab1

C25_MPZL3 - 1.ab1

C25_MPZL3 - 3.ab1

C25_MPZL3 - 4.ab1

C25_SCN1B - 1.ab1

C25_SCN1B - 2.ab1

C25_SCN1B - 3.ab1

C25_SCN1B - 4.ab1

C25-MPZL1 - 1.ab1

C25-MPZL1 - 2.ab1

C25-MPZL1 - 3.ab1

C25-MPZL1 - 4.ab1

C25-MPZL1 - 5.ab1

C25-MPZL1 - 6.ab1

C25-MPZL1 - 7.ab1

C25-MPZL3 - 2.ab1

C25-SCN3B.ab1

JAML-1.fas

JAML-3.fas

MPZL2 MPZL3 JAML.fas

NC_000001.11_MPZ.gbk

NC_000001.11_MPZ_beHAPe.gbk

NC_000001.11_MPZ_Clone25.gbk

NC_000001.11_MPZL1.gbk

NC_000001.11_MPZL1_Clone25.gbk

NC_000011.10_JAML.gbk

NC_000011.10_JAML_BeHAPe.gbk

NC_000011.10_MPZL2.gbk

NC_000011.10_MPZL2_Clone25.gbk

NC_000011.10_MPZL3.gbk

NC_000011.10_MPZL3_Clone25.gbk

NC_000011.10_SCN2B.gbk

NC_000011.10_SCN2B_beHAPe.gbk

NC_000011.10_SCN3B.gbk

NC_000011.10_SCN3B_C25.gbk

NC_000011.10_SCN4B.gbk

NC_000011.10_SCN4B_C25.gbk

NC_000019.10_SCN1B.gbk

NC_000019.10_SCN1B_C25.gbk

SCN2B.fas
