# Supplementary material for: Beta-subunit-eliminated eHAP expression (BeHAPe) cells reveal subunit regulation of the cardiac voltage-gated sodium channel
Source: J Biol Chem. 2023 Aug 6;299(9):105132. doi: 10.1016/j.jbc.2023.105132 (PMC10506104; doi:10.1016/j.jbc.2023.105132)
Supplement: Supporting Figure S1 [file mmc3.pdf]

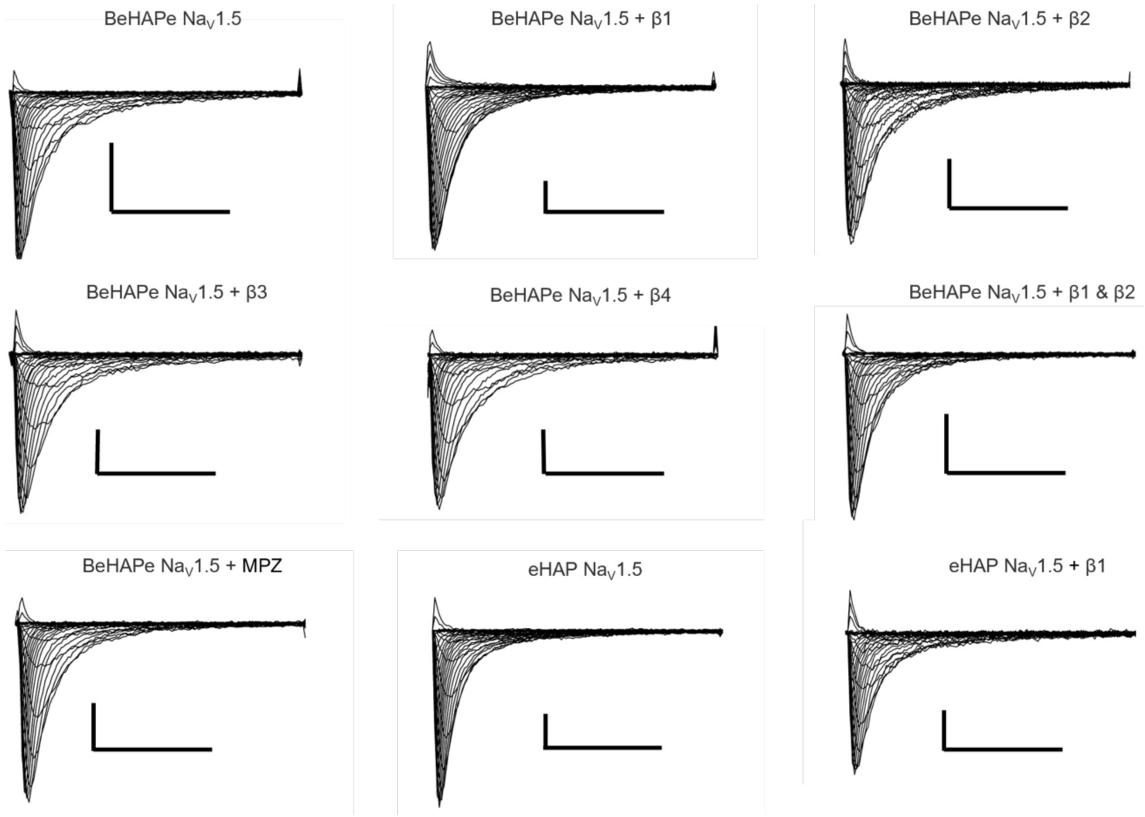

Supplemental Figure 1.
Representative trace families of each experimental condition. All scale bars are 1nA and 10ms.
